# Integrated therapy for HIV and cryptococcosis

**DOI:** 10.1186/s12981-016-0126-7

**Published:** 2016-11-29

**Authors:** Sirawat Srichatrapimuk, Somnuek Sungkanuparph

**Affiliations:** 1 Chakri Naruebodindra Medical Institute, Faculty of Medicine Ramathibodi Hospital, Mahidol University, Samutprakan, Thailand; 2 Division of Infectious Diseases, Department of Medicine, Faculty of Medicine Ramathibodi Hospital, Mahidol University, Bangkok, Thailand

**Keywords:** HIV, Cryptococcosis, Treatment, Integrated therapy

## Abstract

Cryptococcosis has been one of the most common opportunistic infections and causes of mortality among HIV-infected patients, especially in resource-limited countries. Cryptococcal meningitis is the most common form of cryptococcosis. Laboratory diagnosis of cryptococcosis includes direct microscopic examination, isolation of *Cryptococcus* from a clinical specimen, and detection of cryptococcal antigen. Without appropriate treatment, cryptococcosis is fatal. Early diagnosis and treatment is the key to treatment success. Treatment of cryptococcosis consists of three main aspects: antifungal therapy, intracranial pressure management for cryptococcal meningitis, and restoration of immune function with antiretroviral therapy (ART). Optimal integration of these three aspects is crucial to achieving successful treatment and reducing the mortality. Antifungal therapy consists of three phases: induction, consolidation, and maintenance. A combination of two drugs, i.e. amphotericin B plus flucytosine or fluconazole, is preferred in the induction phase. Fluconazole monotherapy is recommended during consolidation and maintenance phases. In cryptococcal meningitis, intracranial pressure rises along with CSF fungal burden and is associated with morbidity and mortality. Aggressive control of intracranial pressure should be done. Management options include therapeutic lumbar puncture, lumbar drain insertion, ventriculostomy, or ventriculoperitoneal shunt. Medical treatment such as corticosteroids, mannitol, and acetazolamide are ineffective and should not be used. ART has proven to have a great impact on survival rates among HIV-infected patients with cryptococcosis. The time to start ART in HIV-infected patients with cryptococcosis has to be deferred until 5 weeks after the start of antifungal therapy. In general, any effective ART regimen is acceptable. Potential drug interactions between antiretroviral agents and amphotericin B, flucytosine, and fluconazole are minimal. Of most potential clinical relevance is the concomitant use of fluconazole and nevirapine. Concomitant use of these two drugs should be cautious, and patients should be monitored closely for nevirapine-associated adverse events, including hepatotoxicity. Overlapping toxicities of antifungal and antiretroviral drugs and immune reconstitution inflammatory syndrome are not uncommon. Early recognition and appropriate management of these consequences can reinforce the successful integrated therapy in HIV-infected patients with cryptococcosis.

## Background

Cryptococcosis is an important opportunistic infection among HIV-infected patients particularly in sub-Saharan Africa and in South and Southeast Asia. It is estimated that more than 600,000 deaths each year globally are from cryptococcosis [1]. The most common clinical presentation is cryptococcal meningitis. Pulmonary and other presentations are less common, and disseminated infection may occur [2]. Although the widespread availability of antiretroviral therapy (ART) has substantially reduced cryptococcosis prevalence worldwide, it is still a major problem in developing countries. Without appropriate treatment, cryptococcosis is fatal. Early diagnosis and treatment is the key to treatment success. Treatment of cryptococcosis consists of three main aspects: antifungal therapy, intracranial pressure management for cryptococcal meningitis, and restoration of immune function with ART. Optimal integration of these three aspects is crucial to achieving successful treatment and reducing the mortality.

ART has proven to have a great impact on survival rates among HIV-infected patients with cryptococcosis [3, 4]. The relapse rate of cryptococcal meningitis after antifungal therapy is also substantially reduced in patients receiving ART [3]. However, the time to start ART in HIV-infected patients with cryptococcosis has to be carefully considered. A randomized trial has recently demonstrated that deferring ART for a specific duration after the start of antifungal therapy improved survival rates among patients with cryptococcal meningitis, as compared with immediate initiation of ART [5]. This improved survival associated with deferring ART was observed in patients with advanced HIV infection. An integrated therapy of both cryptococcosis and HIV based on the current evidence of studies from both diseases can yield better survival. This article focuses on the integrated therapy for HIV-infected patients with cryptococcosis and details regarding diagnosis and treatment of cryptococcosis, initiation of ART, management of drug–drug interaction, overlapping toxicities of antifungal and antiretroviral drugs, as well as cryptococcal immune reconstitution inflammatory syndrome (IRIS).

### Clinical manifestations of cryptococcosis in HIV-infected patients

Infection by *Cryptococcus* is believed to occur mainly after inhalation of desiccated yeast cells or basidiospores into the alveoli. Other proposed portals of entry include gastrointestinal tract, direct inoculation from trauma, and transplantation of an infected organ [6–8]. In HIV-infected patients, dissemination may follow. Alternatively, *Cryptococcus* may initially establish a latent infection within thoracic lymph nodes or a pulmonary granuloma of a healthy host. These dormant viable yeast cells reactivate when the host becomes subsequently immunosuppressed. *Cryptococcus* can infect nearly any organ, but the most common are the central nervous system (CNS) and the lungs. Wide ranges of clinical manifestations and severity have been reported, depending on involved organs, patient’s immune status, and species or strains of the yeast. HIV-infected patients with cryptococcosis frequently present with disseminated disease [9].

Cryptococcal meningitis is the most common form of CNS cryptococcosis. Symptoms and signs include headache, fever, cranial neuropathy, alteration of consciousness, lethargy, memory loss, and meningeal irritation signs [2, 9]. These signs and symptoms typically have a subacute onset, although acute and chronic onset can also be observed. Classic signs of meningeal irritation can present in a minority of patients [9]. Increased intracranial pressure often complicates cryptococcal meningitis and contributes significantly to the morbidity and mortality [10–12]. Patients with cryptococcoma usually have focal neurological deficits, blindness, seizures, as well as signs of increased intracranial pressure [13, 14]. Other reported neurological complications include cerebral infarction from cerebral vasculitis, and venous sinus thrombosis [15, 16].

Pulmonary cryptococcosis has clinical manifestations varying from asymptomatic colonization to acute respiratory distress syndrome (ARDS). Common signs and symptoms in HIV-infected patients are cough, dyspnea, pleuritic chest pain, and constitutional symptoms, such as fever, malaise, and weight loss [17–21]. Some patients may also have hemoptysis and hypoxemia. In HIV-infected patients, pulmonary cryptococcosis is more severe and has a more acute onset than that in other hosts. There is a higher risk of progression, with ARDS occasionally occurring [18]. Furthermore, pulmonary cryptococcosis in HIV-infected patients is usually a clinical manifestation of disseminated infection.

Cutaneous cryptococcosis is characterized by various types of skin lesions, including papules, plaques, purpura, nodules, ulcers, cellulitis, abscesses, and sinus tracts [22]. In AIDS patients, it commonly presents as multiple painless papules with central ulceration, which resembles the lesions caused by *Molluscum contagiosum, Histoplasma capsulatum, Talaromyces marneffei*, and *Coccidioides immitis* [22]. Similar to pulmonary cryptococcosis, skin lesions are often parts of disseminated disease.

Ocular manifestations of cryptococcosis are occasionally observed. Photophobia, diplopia, papilledema, ocular palsy, and temporary or permanent visual loss have been reported [23–25]. These signs and symptoms are due to inflammation from direct invasion of the yeast or increased intracranial pressure. Visual loss with a rapid onset is usually a result of the former process, while a delayed onset is a result of the latter process [25]. Although recovery of vision loss related to cryptococcal meningitis is not predictable, dramatic improvement with corticosteroids has been reported in patients with vision loss from immune reconstitution inflammatory syndrome (IRIS) [25].

Cryptococcal lymphadenitis is in the differential diagnosis list of HIV-infected patients presenting with lymphadenopathy. Cervical lymph nodes are typically the most involved. Cryptococcosis of several other sites has been reported in the literature. These sites include genital and urinary tracts, thyroid, adrenal gland, head and neck, breasts, bone and joints, muscles, endocarditis, myocarditis, pericarditis, vascular graft infection, mycotic aneurysm, gastrointestinal nodules or ulcers, hepatitis, and peritonitis [26–32].

### Diagnosis of cryptococcosis in HIV-infected patients

Laboratory diagnosis of cryptococcosis includes direct microscopic examination, isolation of *Cryptococcus* from a clinical specimen, and detection of cryptococcal antigen. *Cryptococcus* in clinical specimens appears as encapsulated spherical or oval yeasts without pseudohyphae or hyphae. The size of the yeast ranges from 5 to 10 µm. In tissues, the yeast usually shows a large capsule. Occasional narrow-based buddings may be seen [33]. A number of techniques can be employed to visualize the yeast. India ink staining shows a clear halo of a capsule around the yeast within a black background. India ink staining is usually applied to cerebrospinal fluid (CSF) and is not suitable for other specimens, e.g. urine, sputum, or bronchoalveolar lavage. In HIV-infected patients, the sensitivity of this test is over 80%. Centrifugation of CSF may improve the sensitivity of the test [33]. Experience is required to distinguish leukocytes and artefacts from the yeast.

Histopathological examination of biopsies and cytologies can be applied to various tissues, biological fluids, bronchoalveolar lavage, and fine-needle aspirations. Hematoxylin and eosin staining usually reveals eosinophilic or lightly basophilic yeasts with surrounding clear halos of the capsules [33]. Tissue reaction varies from minimal inflammation in highly immunosuppressed host, to a spectrum of granulomatous reactions and fibrosis [33]. A number of special stains may be employed to better visualize *Cryptococcus*. Mucicarmine and Alcian blue stain the fungal capsule. Gomori methenamine silver, periodic acid-Schiff, and calcofluor white stain the fungal cell wall. The yeast is also positive for Fontana-Masson staining, since it produces melanin. This feature can be used to differentiate *Cryptococcus* from *Candida* and *Histoplasma* [33]. Gram stain reveals poorly stained gram-positive yeasts, but is not recommended for *Cryptococcus* identification.

*Cryptococcus* can be cultured from most sites in standard fungal media in the absence of cycloheximide. It can also grow in bacterial media. Colonies appear on solid media as white-to-cream opaque mucoid colonies usually within 3 days. Delayed growth up to 4 weeks may be observed if patients have already received antifungal therapy. Identification of species can be done by biochemical methods, molecular methods, or matrix-assisted laser desorption/ionization time-of-flight (MALDI-TOF) mass spectrometry [34]. Sensitivities of CSF and blood cultures in HIV-infected patients with cryptococcal meningitis are approximately 90 and 50–70%, respectively [35]. Antifungal susceptibility testing is not routinely recommended for initial management of cryptococcosis, but may be considered in relapse and persistent disease [2, 9].

Detection of cryptococcal antigen, which is a component of polysaccharide capsule of the yeast, is a highly sensitive and specific test. Most clinical specimens are CSF and serum. Whole blood and plasma have recently been shown to have comparable sensitivity and specificity [36]. Urine cryptococcal antigen testing has also been proposed as a non-invasive diagnostic test, albeit lower levels of antigen were detected as compared with those in the serum [36]. Further studies are warranted regarding the performance of cryptococcal antigen testing in specimens other than CSF and blood. Currently, available detection methods comprise of latex-agglutination, enzyme immunoassay, and lateral flow assay. All of them showed very high sensitivity and specificity for CSF and serum testing [37]. False negative results may be due to low fungal burden, poorly encapsulated strains, and if latex-agglutination or lateral flow assay is used, prozone/hook effect [35, 38, 39]. Dilution of samples before testing should be performed in case of suspected prozone or hook effect. False positive results of latex agglutination test have been rarely reported in infections caused by *Trichosporon asahii*, *Stomatococcus mucilaginosus*, *Klebsiella* spp., and *Capnocytophaga canimorsus* and agar syneresis fluid [40–43]. Titers of false positive results are usually below 1:8. Cryptococcal antigen testing possesses not only diagnostic value, but also prognostic value. High cryptococcal antigen titers in CSF and serum have shown to be associated with poor outcomes, including death, relapse, treatment failure and IRIS [44–51]. However, the test is not recommended for monitoring after treatment (see below).

For HIV-infected patients suspected of having cryptococcal meningitis/meningoencephalitis, a lumbar puncture should be performed. Besides the CSF examination, this procedure serves to reduce the usually increased intracranial pressure. Brain imaging should be sent before the procedure in patients who have focal neurological deficits or impaired consciousness. Analysis of the CSF generally reveals normal to mild pleocytosis, with a mononuclear predominance, mildly elevated protein levels, and low-to normal glucose levels [52–54]. Normal CSF white blood cell (WBC) counts, protein levels and glucose levels can be occasionally observed. A bedside procedure of India ink staining is simple and helpful. However, given the sensitivity of India ink tests, CSF cryptococcal antigen should also be performed regardless of the India ink staining results. Testing for serum cryptococcal antigen should be considered as well. Positive cryptococcal antigen testing in the CSF provides a strong support for the diagnosis of cryptococcal meningitis/meningoencephalitis, and treatment should be administered. CSF culture for fungus should be sent. Notably, yields of CSF fungal cultures were shown to be higher if a greater amount of CSF was used [55]. A considerable proportion of brain imaging of HIV-infected patients with cryptococcal meningitis/meningoencephalitis may be normal or non-specific [56–59]. Indeed, signs of meningeal inflammation are usually absent in HIV-infected patients with cryptococcal meningitis.

In pulmonary cryptococcosis, chest imaging findings are various and non-specific. Local or diffuse infiltration, nodules, hilar lymphadenopathy, cavitation, and pleural effusion have been described [17, 60–62]. Occasionally, the findings of diffuse infiltrates may be confused with those of *Pneumocystis jiroveci* pneumonia [60]. Given the non-specific nature of imaging, microbiological diagnosis is usually required to establish the diagnosis. There are several agents of pulmonary opportunistic infection in HIV-infected patients, e.g. *Nocardia* spp., *Mycobacterium* spp., *Pneumocystis jiroveci*, and cytomegalovirus. Consequently, in addition to sputum, bronchoalveolar lavage with or without lung biopsy may be required. In HIV-infected patients, pulmonary cryptococcosis may progress to, or be a clinical manifestation of disseminated disease. In fact, those with pulmonary cryptococcosis often already have CNS involvement at diagnosis. Serum cryptococcal antigen, blood culture, and lumbar puncture with CSF examination should be performed [2]. Cryptococcus involvement of other organs, e.g. skin, and lymph nodes, usually need histopathological examination. Investigation for systemic involvement should also be considered.

HIV-infected patients with CD4 counts <100 cells/µL, especially those not taking ART, are at risk of cryptococcosis. Serum cryptococcal antigen can be detected before the onset of symptoms. Screening of such patients and preemptive treatment of those whose test is positive was shown to improve outcomes and was cost-effective [63–65].

### Integrated therapy of HIV and cryptococcosis: general concept

Without appropriate treatment, cryptococcosis in HIV-infected patients has a very high mortality rate [66, 67]. The two diseases, cryptococcosis and HIV, must be managed simultaneously. Overall mortality in HIV-infected patients is high during the first few months of treatment [66, 68]. Causes of mortality are cryptococcosis-related, other life-threatening opportunistic infections, and/or cryptococcal IRIS. Managing the two diseases more effectively during this critical period is essential to improve the patients’ survival and quality of life. Although both cryptococcosis and tuberculosis occur in HIV-infected patients with advanced HIV disease, integrated therapies for each disease and HIV are different [69]. The time to start ART in HIV-infected patients with cryptococcosis has to be carefully considered. A randomized trial has recently demonstrated that deferring ART until 5 weeks after the start of antifungal therapy improved survival rates among patients with cryptococcal meningitis, as compared with initiating ART at 1–2 weeks [5]. Therefore, optimally integrated therapy of HIV and cryptococcosis is the key to improving survival. There are three main aspects of integrated therapy for HIV and cryptococcosis: (1) antifungal therapy, (2) intracranial pressure management for cryptococcal meningitis, and (3) ART to restore immune function.

Integration of treatment for both cryptococcosis and HIV using a single facility, a single health care provider, and delivering care for both diseases is a successful model [3, 4, 70]. The advantages include providing optimal timing for initiation of ART in HIV-infected patients with cryptococcosis, holistic evaluation of the patients, and practical management when patients encounter drug–drug interaction or adverse drug effects. In addition, this model in resource-limited settings is also more feasible to set up, maintain, and train the healthcare providers. Interventions for improving adherence and social support can also better reinforce this approach.

### Treatment of cryptococcosis in HIV-infected patients

#### Antifungal therapy

Cryptococcus is susceptible to polyenes, flucytosine, and azoles. Among these drugs, azoles exhibit the least fungicidal activity. Antifungal treatment for cryptococcosis varies according to the disease extent, severity, as well as host immune status. Although there are some clinical distinctions between cryptococcosis due to *C. neoformans* and *C. gattii,* recommended treatment regimens for both species are currently identical. Some experts suggest that a longer duration of induction and consolidation therapy should be used in *C. gattii* infection [71, 72]. Fluconazole monotherapy is recommended for mild-to-moderate pulmonary disease. In contrast, treatment of disseminated disease, severe pulmonary disease, and meningitis/meningoencephalitis consists of three phases: induction, consolidation, and maintenance. A combination of two drugs is preferred in the induction phase. Fluconazole monotherapy is recommended during consolidation and maintenance phases (Table 1) [2].

**Table 1:** Antifungal therapy for cryptococcosis in HIV-infected patients

Meningitis/meningoencephalitis, disseminated disease, severe pulmonary disease^a^	
Induction phase	
Amphotericin B deoxycholate^b^ (0.7–1 mg/kg/day) plus flucytosine (100 mg/kg/day)	2 weeks
Alternative regimens	
Amphotericin B deoxycholate (0.7–1 mg/kg/day) plus fluconazole (800 mg/day)	2 weeks
Amphotericin B deoxycholate^b^ (0.7–1 mg/kg/day)	4–6 weeks
Fluconazole (≥800 mg/day, preferably 1200 mg/day) plus flucytosine (100 mg/kg/day)	6 weeks
Fluconazole (800–2000 mg/day, preferably ≥1200 mg/day)	10–12 weeks
Itraconazole (400 mg/day)	10–12 weeks
Consolidation phase	
Fluconazole (400 mg/day)	8 weeks
Alternative regimens	
Itraconazole (400 mg/day)	8 weeks
Maintenance phase	
Fluconazole (200 mg/day)	≥1 year^c^
Alternative regimens	
Itraconazole (400 mg/day)	≥1 year^c^
Amphotericin B deoxycholate (1 mg/kg/week)	≥1 year^c^
Mild-to-moderate pulmonary disease	
Fluconazole (400 mg/day)	6–12 months

^a^For cerebral cryptococcoma, consider induction treatment for ≥6 weeks and consolidation and maintenance treatment for 6–18 months; for isolated cryptococcal antigenemia, consider (1) induction treatment with oral fluconazole at the dose of 800 mg/day for 2 weeks, then proceed to consolidation and maintenance treatment, or (2) oral fluconazole at the dose of 400 mg/day for 12 months

^b^Liposomal amphotericin B (3–4 mg/kg/day; as high as 6 mg/kg/day) or Amphotericin B lipid complex (5 mg/kg/day) serves as an alternative to amphotericin B deoxycholate, with less nephrotoxicity and infusion reaction

^c^Patients should have suppressed or very low viral load, and have CD4 counts >100 cells/μl for at least 3 months before discontinuing maintenance treatment

Amphotericin B plus flucytosine has been shown to be the most potent and advocated regimen for the induction phase [2, 9, 73–77]. Intravenous amphotericin B deoxycholate should be given at a dose of 0.7–1 mg/kg/day [2, 9]. Liposomal amphotericin B at a dose of 3–4 mg/kg/day or amphotericin B lipid complex at a dose of 5 mg/kg/day can be used as a substitute for amphotericin B deoxycholate, as these formulations cause less nephrotoxicity and infusion reaction, while demonstrating similar, if not better, efficacy [78, 79]. Flucytosine should be used at a dose of 100 mg/kg/day, given in four divided doses. Alternative regimens for the induction phase include amphotericin B plus fluconazole, amphotericin B monotherapy, fluconazole plus flucytosine, and high-dose fluconazole monotherapy [2, 9]. Unfortunately, flucytosine is not available in many countries in resource-limited settings and cannot be used in patients with bone marrow suppression and liver enzyme elevations. Amphotericin B deoxycholate at a dose of 0.7 mg/kg/day plus 800 mg/day of fluconazole might have better efficacy than amphotericin B alone [73, 79–81]. A high fluconazole dosage at 800 mg/day is also associated with high serum and CSF fluconazole concentration and appears to be associated with increased survival and treatment success [82]. Moreover, no additional toxicity was observed [73, 79–81]. If the patient cannot tolerate amphotericin B or the drug is not available, the induction phase can be achieved by fluconazole plus flucytosine, or high-dose fluconazole monotherapy (Table 1) [2, 9]. However, these two regimens have lowered efficacy. As a result, higher doses of fluconazole and longer durations of treatment are suggested [2, 9, 83]. Fluconazole plus flucytosine has proven better than fluconazole monotherapy [84–86].

After the Induction phase of treatment, the consolidation phase commences. Longer durations of induction should be considered if clinical improvement is not evident and/or CSF culture at 2 weeks is still positive [2, 9]. Patients who fail to achieve negative CSF culture after 2 weeks were shown to have higher risks for treatment failure at 10 weeks [87]. Fluconazole is the drug of choice during this phase (Table 1). It is noted that 800 mg/day, rather than 400 mg/day, of fluconazole should be used if amphotericin B plus fluconazole regimen is selected for induction. The consolidation phase continues for at least 8 weeks. Then, the fluconazole dose should be reduced to 200 mg/day during the maintenance phase. As long as the patients’ CD4 cell counts remain low, they are still at high risk for relapse. The maintenance phase can be safely discontinued in patients who have been treated for at least 12 months, have suppressed or very low viral load, and have CD4 counts >100 cells/µL for at least 3 months [88–92].

Other azoles, such as itraconazole, voriconazole, posaconazole, and isavuconazole, also possess anti-cryptococcal activity. However, given the scarcity of studies of clinical efficacy, potential drug interactions, CNS penetration, and bioavailability, they are reserved for refractory cases only [2, 9, 75, 93–99].

Resistance to antifungal drugs was previously rare. However, recent reports described increased MICs of *C. neoformans* isolates to fluconazole and, to a lesser extent, amphotericin B over the past decade [100, 101]. Whether this has an impact on clinical outcomes is not known. Furthermore, clinical breakpoints of *Cryptococcus* spp. to antifungal drugs are not yet established. Some small studies suggested that worse treatment outcomes might be associated with higher MICs to antifungal drugs, although this is still controversial [100–105]. Currently, testing for antifungal susceptibility might be considered only in patients with persistent or relapse disease [2, 9]. An MIC of ≥16 µg/mL for fluconazole may be considered resistant, and alternative treatment is suggested, e.g. intravenous amphotericin B deoxycholate at a dose of 1 mg/kg/day until CSF, blood, and/or other sites are sterile [2]. An MIC of ≥32 µg/mL for flucytosine may be considered resistant, and the induction phase with non-flucytosine-containing regimen may be selected [2].

Cerebral cryptococcoma should be treated with the same regimen used for cryptococcal meningitis, but with an extended duration of therapy (Table 1) [2]. This should also be guided by clinical response and imaging during treatment. The recommended dose of fluconazole during consolidation is 400–800 mg/day [2]. Surgery is rarely necessary, except when other etiologies are suspected and histopathological diagnosis is required, or mass effect is observed. It is noteworthy that an MRI of the brain may not show a decrease in lesion size for many months [106]. Adjunctive treatment with corticosteroids with gradual tapering might be considered in patients with significant perilesional edema [2].

Pulmonary cryptococcosis in HIV-infected patients is frequently a manifestation of disseminated disease. Hence, these patients should be evaluated for CNS involvement and disease dissemination, even if they are asymptomatic. A CSF examination, culture, as well as testing for cryptococcal antigen in the CSF and serum should be performed. Disseminated disease should be treated with the same regimen as that of cryptococcal meningitis [2, 9]. For isolated pulmonary cryptococcosis, treatment varies according to severity. Pulmonary cryptococcosis with severe symptoms and/or diffuse pulmonary infiltrates should be treated with regimens identical to those for disseminated disease and meningitis [2, 9]. For pulmonary cryptococcosis with mild-to-moderate symptoms, a dosage of 6 mg/kg/day of oral fluconazole for 12 months is recommended [2, 9]. Surgical removal and drainage of pleural effusion caused by *Cryptococcus* are rarely required [107].

Similar to pulmonary cryptococcosis, cryptococcal infection of other organs is often a manifestation of disseminated disease, especially in HIV-infected patients. These patients should be evaluated for CNS involvement and disease dissemination as well [2, 9]. For ocular cryptococcosis, combined antifungal agents using systemic amphotericin B with high-eye penetration drugs, such as flucytosine or fluconazole, are recommended [2].

Positive serum cryptococcal antigen has been shown to be associated with subsequent cryptococcal disease [108–110]. Screening for serum cryptococcal antigen in asymptomatic HIV-infected patients with CD4 counts <100 cells/µL and preemptive treatment of those who test positive lead to more favorable outcomes and more cost-effectiveness [63–65, 108, 109, 111–113]. Antifungal regimens have been proposed [9, 114] although sparse data exist: (1) oral fluconazole at the dose of 800 mg/day for 2 weeks, followed by 400 mg/day for 8 weeks, and 200 mg/day thereafter until CD4 count is >200 cells/µL [65], or (2) oral fluconazole at the dose of 400 mg/day for 1 year. HIV-infected patients with serum antigen titer ≥1:512 may be treated as CNS disease [2].

Pregnant women with cryptococcosis represent a special population. Amphotericin B can be used safely without risk of teratogenicity in humans (FDA pregnancy category B) [2, 9, 115]. Use of other antifungal agents, however, should be considered when the benefits outweigh fetal risks. Flucytosine is classified as category C by the FDA. Although animal studies showed teratogenic effect of flucytosine, limited studies in humans showed no adverse fetal outcomes after exposure [112]. Fluconazole is classified as category D. There are few reports of fetal anomalies in pregnant women who had received ≥400 mg/day of fluconazole [116]. Use of fluconazole, as well as other azoles, is thereby discouraged during pregnancy, especially in the first trimester [2, 9, 112]. Neonates born to patients receiving amphotericin B at delivery should be evaluated for renal function and electrolytes [9].

#### Intracranial pressure management for cryptococcal meningitis

In cryptococcal meningitis, intracranial pressure rises along with CSF fungal burden and is associated with morbidity and mortality [10–12]. Increased intracranial pressure may be more prominent in cerebral cryptococcoma. Aggressive control of intracranial pressure should be done [2, 9]. Management options include therapeutic lumbar puncture, lumbar drain insertion [117], ventriculostomy, or ventriculoperitoneal shunt. Therapeutic lumbar puncture is usually selected in most cases. CSF opening pressure should be measured before treatment, and therapeutic lumbar punctures to achieve closing pressure below 20 cm H_2_O or 50% of initial opening pressure are recommended [2, 9]. Brain imaging before the procedure should be considered for patients with alteration of consciousness and/or focal neurological deficits. Lumbar puncture should be performed whenever symptoms of increased intracranial pressure arise. Persistently increased intracranial pressure should be managed by daily lumbar puncture until symptoms abate and normal opening pressure is obtained for >2 days [2, 9]. Interestingly, a recent study showed far less deaths in patients who received at least one therapeutic lumbar puncture, compared to those who did not, regardless of baseline opening pressure [118]. This finding underscores the importance of therapeutic lumbar puncture. Failure to control intracranial pressure with lumbar puncture warrants the need of lumbar drain insertion, ventriculostomy, or CSF shunt placement. This can be performed without a need for CSF sterilization before the procedure [2, 9]. Patients who experience increased intracranial pressure as a manifestation of IRIS should be managed alike.

Medical treatment such as corticosteroids, mannitol, and acetazolamide are ineffective and should not be used [2, 9]. A recent randomized controlled trial showed that routine use of corticosteroids in cryptococcal meningitis might do harm to the patients. This study was terminated prematurely, because there was no difference in mortality or the rate of IRIS between the two groups at 10 weeks [119]. Additionally, the use of steroids was associated with higher risks of disability, higher adverse events, and reduced sterilizing power of amphotericin B plus fluconazole during the induction phase. Therefore, its use is limited to patients with CNS IRIS and cerebral cryptococcoma with significant edema [2, 9]. Table 2 summarizes intracranial pressure management for cryptococcal meningitis.

**Table 2:** Intracranial pressure management for cryptococcal meningitis in HIV-infected patients

Meningitis/meningoencephalitis
Aggressive control of intracranial pressure^a^
Management options
Therapeutic lumbar puncture^b^ (usually selected in most cases)
Lumbar drain insertion
Ventriculostomy
Ventriculoperitoneal shunt^c^
Medical treatment i.e. corticosteroid^d^, mannitol, and acetazolamide are ineffective
Cryptococcoma
Management as in meningitis/meningoencephalitis
Corticosteroids may be used in cryptococcoma with significant brain edema
Cryptococcal IRIS
Management as in meningitis/meningoencephalitis
Corticosteroids may be used in severe IRIS

^a^Brain imaging before the procedure should be considered for patients with alteration of consciousness and/or focal neurological deficits. Lumbar puncture should be performed whenever symptoms of increased intracranial pressure arise. Persistently increased intracranial pressure should be managed by daily lumbar puncture until symptoms abate and normal opening pressure is obtained for >2 days

^b^Therapeutic lumbar punctures to achieve closing pressure below 20 cm H2O or 50% of initial opening pressure are recommended

^c^Can be performed without a need for CSF sterilization before the procedure

^d^The use of steroids was associated with higher risks of disability, higher adverse events, and reduced sterilizing power of amphotericin B plus fluconazole during the induction phase

#### Treatment monitoring and treatment failure of cryptococcosis

After appropriate treatment, clinical improvement should be observed, usually within 2 weeks. In patients with cryptococcal meningitis, increased intracranial pressure usually resolves. Follow-up CSF cultures gradually turn negative. It is recommended by several experts that a CSF culture at 2 weeks should be sent to determine CSF sterility after induction phase [2, 9]. A persistently positive CSF culture at 2 weeks after treatment is associated with morbidity and mortality, higher risk for treatment failure at 10 weeks, relapse, and paradoxical IRIS [76, 87, 120–123]. Serial CSF quantitative cultures have been used as a determinant of fungicidal activity of given treatment regimens, and have been shown to be correlated with morbidity and mortality [9, 73, 75–77, 84, 124]. However, a quantitative culture is rarely done merely for clinical purposes.

Management of patients who fail to achieve CSF sterility at 2 weeks awaits more data, but experts suggest induction therapy for another 2 weeks and a follow-up CSF culture obtained [2, 9]. Nonetheless, the CSF cultures in some of these patients appear to become negative later with continued consolidation therapy [77, 121, 122, 125]. Of note, despite a good correlation between quantitative CSF cultures and cryptococcal antigens at baseline, their kinetics of clearance after treatment differ [126]. CSF cryptococcal antigens may persist for an extended period even if the culture is negative. Using cryptococcal antigen titer to make decisions during therapy is of limited value and is not advisable [2, 9, 127, 128]. Likewise, continued presence of yeasts visualized by India ink does not indicate treatment failure or disease relapse.

Persistence is arbitrarily defined as lack of clinical improvement and continued positive cultures after 2–4 weeks of appropriate therapy [2, 9]. In such a case, it should be assessed whether the treatment regimen, intracranial pressure management, as well as treatment adherence are optimal. Potential drug interaction should be sought. Brain imaging might be considered to rule out cryptococcoma. Although rare, concomitant opportunistic infections or malignancy is also possible, given the usually severe immunocompromised status of the patients [129] and proper investigations should be sent. In addition, MICs of the persistent isolate should be checked and compared with those of the original isolate [2, 9]. A ≥3-dilution increase suggests development of drug resistance [2]. Re-induction, typically with combined antifungal agents, should be administered. A standard regimen should be used if a regimen with lowered efficacy was previously given. Higher dose and/or longer course (4–10 weeks) may be considered [2, 9]. Although more clinical studies are required, MICs may be used to guide treatment regimen selection, as mentioned above. There are few case reports that describe a successful outcome of a combination of multiple antifungal agents, including newer azoles, or adjunctive treatment with recombinant interferon-γ1b [95, 128, 130, 131].

Relapse is defined as a recurrence of symptoms, after an initial resolution, with a positive CSF culture after ≥4 weeks of treatment [2, 9]. It remains challenging for clinicians taking care of patients who develop recurrent symptoms, as this may be secondary to several possible etiologies. These include disease relapse, paradoxical IRIS, new opportunistic conditions, drug toxicity, or a combination thereof. It is crucial to determine the etiology, as each requires a distinct management strategy. However, it is difficult to distinguish by clinical signs and symptoms of the patients alone. Thus, further investigations both for *Cryptococcus* spp. and other potential pathogens are warranted. Disease relapse may be more likely if the patient has not yet received ART or has virologic failure. Patients who received inadequate induction treatment and/or had poor adherence to consolidation/maintenance therapy are also at high risk for disease relapse. Relapse was more frequent in cohorts using induction therapy with only fluconazole monotherapy [45, 88]. Among patients without these clues, discriminating between disease relapse and IRIS is difficult. Limited data showed that patients with disease relapse returned later, were less likely to receive ART, had less CD4 count increases and less viral load declines in response to ART, and had lower CSF opening pressure than those with IRIS [132]. Another recent study stated that no clinical features differentiated relapse from IRIS, but demonstrated lower CSF WBC counts and lower CSF interferon-γ, TNF-α, IL-4, IL-9, IL-12, and IL-17 [52]. Yet another study showed that serum CRP levels were all normal in patients with disease relapse, while those of patients with IRIS were elevated in 76% [44]. After all, a CSF culture remains the gold standard for diagnosing disease relapse [2]. Of note, antifungal treatment might delay the yeast growth, and it may take up to 4 weeks to obtain the final result. In practice, induction therapy should be reinstituted, while CSF culture result is pending. If the disease relapse is confirmed, experts recommend management similar to that of persistent—re-induction, probably with a higher dose and longer course, and selecting the regimen according to MICs of the yeast [2]. Newer azoles may be considered. If the isolate is fluconazole-susceptible and poor-adherence is a problem, prior suppressive doses of fluconazole may be used [2]. Like newly diagnosed patients, those with persistence or relapse should be monitored for potential drug toxicity and appropriate intracranial pressure management should be exercised.

### ART in HIV-infected patients with cryptococcosis

ART constitutes an essential component in the management of AIDS-related cryptococcosis. Most patients are antiretroviral-naïve at the time of diagnosis of cryptococcosis. Delayed ART initiation places the patients at risk for other opportunistic infections. However, too early ART initiation increases the risk of developing IRIS. Consequently, the time as to when ART should be commenced is crucial. Delayed initiation of ART until at least after completion of induction therapy and possibly until after completion of consolidation therapy, has been recommended [2]. A recent landmark randomized clinical trial conducted in Africa demonstrated that 26-week mortality was significantly higher in patients who received early ART (1–2 weeks after antifungal treatment) compared with those who received delayed ART (5 weeks after antifungal treatment). There was no significant difference of cryptococcal IRIS between the two groups, although it was proposed that early unrecognized CNS IRIS might account for the mortality difference [5, 133]. For patients with asymptomatic cryptococcal antigenemia, optimal timing of ART initiation has not been established. Currently, deferred initiation of ART until 5 weeks of antifungal therapy in this population is also suggested.

In general, any effective ART regimen is acceptable. There is no data regarding the benefit of one certain ART regimen over another. Amphotericin B does not affect the CYP450 enzyme system; therefore, pharmacokinetic interactions between amphotericin B and most antiretroviral agents are not expected [134]. However, at the time of ART initiation in HIV-infected patients with cryptococcosis, antifungal therapy is in the consolidation phase with fluconazole. When choosing an initial ART regimen, one must consider drug–drug interaction between fluconazole and antiretroviral agents. With protease inhibitors (PIs), fluconazole may potentially increase PI concentrations, primarily due to CYP3A4 inhibition. However, these effects do not appear to be clinically significant and fluconazole may be coadministered without dose adjustment [134].

As a relatively modest inhibitor of CYP3A4, fluconazole has the potential to increase concentrations of NNRTIs. No significant interactions have been observed with efavirenz [134]. A clinical trial of ART initiation in advanced HIV-infected patients with cryptococcal meningitis has shown that an efavirenz-based regimen is effective, safe, and well-tolerated [135]. Concomitant use of fluconazole and nevirapine has the most potential clinical relevance. A cohort study to compare the adverse events after the initiation of a nevirapine-based regimen in 686 HIV-infected patients who received and did not receive fluconazole has shown that there were no significant differences of clinical hepatitis, elevated aminotransferase, or skin rashes between groups [136]. In another retrospective study of 122 HIV-infected patients who received nevirapine, those also taking fluconazole 200 or 400 mg/day had nevirapine Cmin 76% higher, compared to those not taking fluconazole. One patient on fluconazole developed clinical hepatitis. There was no difference in terms of 36-week antiretroviral efficacy between the two groups [137]. Nevirapine and fluconazole should be concomitantly used with caution, and patients monitored closely for nevirapine-associated adverse events, including hepatotoxicity. For rilpivirine, there is no clinical significance of drug interaction with fluconazole [133]. Although potential drug interactions between antiretroviral agents and amphotericin B, flucytosine, and fluconazole are minimal, care must be exercised if other azoles are used. Table 3 summarizes ART in HIV-infected patients with cryptococcosis.

**Table 3:** ART in HIV-infected patients with cryptococcosis

Time to start ART
Deferred until 5 weeks after the start of antifungal therapy
ART regimen
Consider drug–drug interactions between fluconazole and antiretroviral agents
Fluconazole may potentially increase PI concentrations, but no clinical significance
Fluconazole has the potential to increase NNRTI concentration
No significant interactions with efavirenz
Nevirapine has the most potential clinical relevance^a^
No significant interactions with rilpivirine
Monitor closely for nevirapine-associated adverse events, including hepatotoxicity

^a^HIV-infected patients who received nevirapine, those also taking fluconazole 200 or 400 mg/day had nevirapine Cmin 76% higher, compared to those not taking fluconazole

### Overlapping toxicities of antifungal and antiretroviral drugs

Amphotericin B has multiple adverse effects, e.g. nephrotoxicity, anemia, electrolyte abnormalities, and infusion reactions [138]. Therefore, careful monitoring of renal function, electrolytes, and complete blood counts should be done. In sub-Saharan Africa, standardized electrolyte supplementation and fluid management for patients treated with amphotericin B deoxycholate have been associated with improved early survival [139]. Liposomal amphotericin B or amphotericin B lipid complex may be considered in patients with high risks for drug toxicity. Moreover, modification of an amphotericin B deoxycholate infusion regimen to 24 h-continuous infusion might lower nephrotoxicity without affecting fungicidal activity, although data is limited [140, 141].

Regarding overlapping toxicity with antiretroviral drugs, both amphotericin B and tenofovir can cause nephrotoxicity. Concomitant use of these agents should be considered with caution. In a cohort study of 222 HIV-infected patients, prior exposure to amphotericin B was found to be a risk factor for nephrotoxicity [142]. In contrast, a recent study showed that tenofovir use in patients receiving induction treatment of cryptococcal meningitis with amphotericin B was not associated with short term nephrotoxicity, as measured by serum creatinine at 4 weeks [143]. Nevertheless, close monitoring of serum creatinine and urinalysis is recommended for concomitant or sequential use of amphotericin B and tenofovir. If renal function is compromised, tenofovir dose should be adjusted accordingly.

Anemia represents a common toxicity with amphotericin B therapy in HIV-infected patients with cryptococcal meningitis. A recent study has demonstrated that amphotericin B-induced anemia was mostly transient and did not impact mortality [144]. Amphotericin B may also cause anemia when used with zidovudine, due to additive bone marrow suppression [133]. Close monitoring of the complete blood count is recommended during therapy. Flucytosine use is associated with bone marrow suppression and liver toxicity. Adjustment of flucytosine dose according to creatinine clearance is essential.

Fluconazole may cause prolongation of the QT interval either directly or by inhibiting the hepatic metabolism of other QT-prolonging agents. A clinical trial to assess QT intervals in 141 HIV-infected patients with cryptococcal meningitis has demonstrated that there were no differences of QTc interval prolongation between those that received and did not receive fluconazole 800 mg/day in addition to amphotericin B in the induction phase of treatment [145]. However, high trough concentration of fluconazole appears to be associated with a trend towards increased risk of QTc prolongation at day 7. Therefore, electrocardiogram monitoring of patients taking high-dose fluconazole should be performed and physicians should be aware of other potential risk factors for QTc prolongation, particularly hypokalemia.

### Immune reconstitution inflammatory syndrome (IRIS)

Immune reconstitution inflammatory syndrome (IRIS) is characterized by clinical deterioration with symptoms and signs of inflammation, resulting from exaggerated host immune responses against pathogens or antigens during immune reconstitution. IRIS can occur after ART initiation, re-initiation, or switching to a more active regimen following virological failure. It has a wide range of clinical manifestations as well as severity. IRIS symptoms often mimic those of infections, and represent a diagnostic challenge for clinicians.

IRIS can be classified into paradoxical IRIS and unmasking IRIS. The former describes a worsening, after an initial clinical improvement of a pre-existing infectious process following ART initiation, while the latter describes a subclinical disease that becomes clinically apparent after ART introduction. Consensus definitions of cryptococcal IRIS have been proposed by the International Network for the Study of HIV-associated IRIS (INSHI) [146].

Paradoxical cryptococcal IRIS has been reported in 8–49% of patients with known cryptococcosis at ART initiation [146]. The reported manifestations vary from relapsing aseptic meningitis, increased intracranial pressure, intracranial cryptococcomas/abscess, spinal cord abscess, focal neurological deficits, lymphadenopathy, pneumonia, soft-tissue disease, eye disease, fever, and a multifocal disease [146–149]. It is noteworthy that IRIS can present in organs that were not initially identified as infected. The reported onset after ART initiation also varies widely, from 4 days to 3 years. The median onsets of IRIS after ART initiation from prospective cohorts are within 10 weeks [146–149]. In CNS IRIS, a CT scan or MRI of the brain may reveal leptomeningeal or choroid plexus gadolinium enhancement, diffuse edema, linear perivascular enhancement in the sulci, or communicating hydrocephalus. This is unlike that of ART-naïve cryptococcal meningitis, which often shows minimal inflammation [56, 150–152]. Meningoradiculitis, enhancing cortical lesions in the cortex compatible with cryptococcomas, and enhancement of the Virchow-Robin spaces can also be seen [56, 150–152].

Pathogenesis of IRIS remains to be further elucidated and is currently an active area of research [153–155]. A number of investigators compared clinical characteristics as well as laboratory results of patients who did and did not later develop IRIS, in order to establish risk factors. The reported risk factors include low baseline CD4 cell counts, high baseline viral load, early ART initiation after antifungal therapy, a poor baseline CSF inflammation (as judged by low CSF protein and WBC counts), different baseline levels of certain CSF cytokines, high baseline certain serum cytokines, high fungal burden, CSF culture positivity at 2 weeks or at ART initiation, substantial CD4 count increase after ART, and rapid viral load decrease after ART [46, 122, 155–160]. However, these studies yielded conflicting results, and some factors require sophisticated instruments. Such factors might help in the prediction, but could not be used as diagnostic criteria. Currently, the proposed INSHI criteria serve to guide the diagnosis of IRIS [146].

Cryptococcal paradoxical IRIS is a diagnosis of exclusion. Patients with clinical deterioration should be initially managed similarly to those with suspected relapse disease, since other possible etiologies of clinical deterioration require distinct management as mentioned above. Shelburne et al. showed that patients with paradoxical IRIS returned later, were more likely to receive ART, had more CD4 count increases and more viral load declines in response to ART, and had higher CSF opening pressure than those with relapse disease [131]. Higher CSF WBC counts and certain CSF cytokines, as well as higher serum CRP were observed in other studies [44, 52]. Of note, the guideline recommends testing for viral load, but does not strictly require it to make the diagnosis. It is deemed that current ART regimens typically have excellent virological response if the patient has good ART adherence [146]. CD4 cell count is also not included in the INSHI criteria [146]. This is due to the fact that paradoxical IRIS was reported in patients with minimally increased CD4 counts after ART initiation [52].

Management of paradoxical cryptococcal IRIS is based on data from case reports and expert opinions. ART and antifungal agent(s) continuation are generally recommended [2, 9]. For mild symptoms, expectant management is reasonable. For severe symptoms of IRIS, corticosteroids [149, 161, 162], NSAIDs [163], and other immunomodulating agents, e.g. hydroxychloroquine [164], thalidomide [165] and adalimumab [166] have been used successfully. CNS IRIS with increased intracranial pressure might be treated with corticosteroids at 0.5–1.0 mg/kg/day of prednisone equivalent or higher for severe CNS signs and symptoms, followed by a subsequent tapering dose in 2–6 weeks [2]. Durations may be adjusted on a case-by-case basis. Aggressive control of intracranial pressure is also critical.

In the unmasking IRIS, the symptoms arise after ART initiation [167, 168]. Given the difficulty of differentiating IRIS from progression of untreated subclinical infection, the term “ART-associated cryptococcosis” was coined by the consensus definition to include both processes [146]. It occurred in 0.2–1.6% of patients without evidence of cryptococcosis before starting ART [146]. Much higher incidences have been observed in patients with subclinical cryptococcal antigenemia who did not receive antifungal therapy [146]. Like paradoxical IRIS, it can manifest as meningitis, CNS complications, skin and soft-tissue disease, lymphadenitis, lung disease, and a disseminated disease [146]. The reported onset of symptoms span a few days to several months. Severe illness usually develops over a few days after onset [146]. The proposed INSHI definitions are currently used. Management of this group of patients should not differ from newly diagnosed cryptococcosis in HIV-infected patients without ART [153].

## Conclusions

The mortality in HIV-infected patients with cryptococcosis is high during the first few months of treatment. Managing the two diseases more effectively during this critical period is essential to improve the patients’ survival and quality of life. The time to start ART in HIV-infected patients with cryptococcosis has to be deferred until 5 weeks after the start of antifungal therapy. Integrated therapy of HIV and cryptococcosis including antifungal therapy, intracranial pressure management for cryptococcal meningitis, and ART to restore immune function is the key to success. Integration of care for both cryptococcosis and HIV using a single facility and a single health care provider is a model to deliver integrated therapy for both diseases and manage overlapping toxicities of antifungal and antiretroviral drugs and IRIS more effectively.
